# Two-Stage Pedestrian Detection Model Using a New Classification Head for Domain Generalization

**DOI:** 10.3390/s23239380

**Published:** 2023-11-24

**Authors:** Daniel Schulz, Claudio A. Perez

**Affiliations:** 1 Department of Electrical Engineering, and Advanced Mining Technology Center, Universidad de Chile, Santiago 8370451, Chile; dschulz@uchile.cl; 2 IMPACT, Center of Interventional Medicine for Precision and Advanced Cellular Therapy, Santiago 7620086, Chile

**Keywords:** pedestrian detection, domain generalization, object detection, triplet loss, two-stage detection

## Abstract

Pedestrian detection based on deep learning methods have reached great success in the past few years with several possible real-world applications including autonomous driving, robotic navigation, and video surveillance. In this work, a new neural network two-stage pedestrian detector with a new custom classification head, adding the triplet loss function to the standard bounding box regression and classification losses, is presented. This aims to improve the domain generalization capabilities of existing pedestrian detectors, by explicitly maximizing inter-class distance and minimizing intra-class distance. Triplet loss is applied to the features generated by the region proposal network, aimed at clustering together pedestrian samples in the features space. We used Faster R-CNN and Cascade R-CNN with the HRNet backbone pre-trained on ImageNet, changing the standard classification head for Faster R-CNN, and changing one of the three heads for Cascade R-CNN. The best results were obtained using a progressive training pipeline, starting from a dataset that is further away from the target domain, and progressively fine-tuning on datasets closer to the target domain. We obtained state-of-the-art results, $MR^{-2}$ of 9.9, 11.0, and 36.2 for the reasonable, small, and heavy subsets on the CityPersons benchmark with outstanding performance on the heavy subset, the most difficult one.

## 1. Introduction

In the last decade, deep learning has enabled significant progress in a variety of applications including object detection [1,2], face recognition [3], iris recognition [4], genetic algorithms applied to CNNs [5,6], rock lithological classification [7], trademark image retrieval [8], and semantic segmentation [9], among others. Pedestrian detection is one of the key tasks in computer vision, for which several models have been developed in the past few years [10,11,12,13,14,15,16,17,18,19]. The performance has shown a steady improvement over time, especially with the boom of deep-learning-based methods, with certain benchmarks approaching human performance [20], e.g., the Caltech benchmark [21]. Many real-world applications require high performance on pedestrian detection, e.g., autonomous driving, robotic navigation, video surveillance, action recognition, and tracking [22,23,24]. In autonomous driving, a robust pedestrian detection method is a key element to develop. Pedestrians tended to suffer more injuries when a crash occurred between vehicles and pedestrians. According to the National Highway Traffic Safety Administration, traffic accidents in the United States generated 7388 pedestrian fatalities, and 60,577 pedestrians were injured in the year 2021 [25]. In Europe, 3608 pedestrian fatalities were reported in 2020, this being 19% of the total road fatalities [26].

The nature of the possible applications involving pedestrian detection makes it necessary to have performance characterized by high accuracy and real-time operation [27]. Some of the main challenges for pedestrian detection methods are that the individuals in the images present different scales, several occlusions, and various aspect ratios, among others [28].

The pedestrian detection problem could be viewed as a sub-problem of the broader object detection problem, and, therefore, many methods can be adapted to detect pedestrians instead of generic objects [29]. In the deep learning approaches, two main methods have been used for object detection: one-stage approaches, such as SSD [30] and YOLO [31], and two-stage approaches, with methods such as Faster R-CNN [2] and Cascade R-CNN [32]. A two-stage object detector includes an intermediate task of generating region proposals, and then, an object classification for each region proposed [32]. In general, one-stage methods are faster than two-stage methods; however, two-stage methods achieve more robust performance [32]. The pedestrian detection task, however, has its own challenges, not shared with the general object detection task. For example, the hard negative instances from background regions usually lead to confusion when detecting pedestrians [33].

The ability of the current state-of-the-art (SOTA) methods to perform with high performance on cross-dataset testing is a problem that has not yet been solved. Hasan et al. [20] showed that the current pedestrian detection methods are unsuccessful when the domain is changed, diminishing performance results when evaluated in cross-dataset scenarios.

In this context, domain change or domain shift is defined as the problem that arises when a distribution shift occurs between a set of training (source) data and a set of test (target) data. This problem is caused by most of the statistical learning methods relying on the assumption that both the source and target data are independent and identically distributed, while ignoring out-of-distribution scenarios that are commonly encountered in practice. This leads to a performance drop when an algorithm trained only with source data is tested on an out-of-distribution target domain. This problem has limited the deployment of large-scale models [34]. Domain generalization is a machine learning problem in which the model learns from labeled training data across related tasks and then is expected to generalize to a future prediction task without access to labeled data [35]. This concept was introduced to address the challenges of domain shift and a lack of target data. The objective is to train a model using data from one or more related but distinct source domains so that it can generalize to perform well and effectively in any out-of-distribution target domain [34].

Our proposed method intends to improve some of the limitations mentioned, relying explicitly on approaches for domain adaptation, such as the triplet loss function. This family of loss functions has been used successfully in other computer vision tasks, e.g., face recognition [3]. Here, we use triplet loss as an additional loss for the classification head, after the region of interest (ROI) extraction, in a two-stage pedestrian detector approach. We use this triplet loss function alongside the traditional classification and bounding box regression losses.

The main contribution of this study is a novel approach to pedestrian detection: the development of a new classification head for two-stage object detectors that incorporates the triplet loss function, to complement the classification and bounding box regression losses, with the objective of enhancing domain generalization capabilities in pedestrian detection tasks. The addition of triplet loss resulted in a new combined loss, that enhanced the feature compactness of pedestrian samples, thereby improving object detection performance relative to SOTA. This head is used at the final stage, being applied to the embeddings generated by the ROI extractor. According to our literature review, the proposed approach has not been used for the pedestrian detection task. One of the main goals of the proposed method is increasing performance on cross-dataset scenarios by maximizing the inter-class distance explicitly, and minimizing the intra-class distance, when a margin term is used to determine the decision boundary between positive and negative pairs. In this way, pedestrians coming from different domains (datasets) are clustered together in the feature space. Another important contribution is achieving improved results, relative to those of the SOTA, for the CityPersons benchmark, for the hardest partition available, using cross-training with a different dataset, i.e., not trained explicitly on any partition of the target dataset, and applied to a complex real-world dataset. Our proposed head could be used as a new direction for improving cross-dataset performance in other pedestrian detectors with compatible architectures, or other object detection tasks, considering real-world applications such as autonomous driving and video surveillance.

## 2. Related Work

The early approaches to pedestrian detection, and in general for object detection, used sliding windows over all the scales and locations [36,37,38]. One of the first methods using this approach was proposed by Papageorgiou and Poggio [37], who used a combination of multiscale Haar wavelets and Support Vector Machines (SVM). The work of Viola and Jones [38] uses the concept of integral images, aimed at speeding up the Haar features computation, and then applies a cascade structure for efficient detection based on AdaBoost classifiers. Dalal and Triggs [39] used features based on the Histogram of Oriented Gradients (HOG), and SVM for human detection, outperforming existing intensity-based features. Dollár et al. [40] proposed Aggregate Channel Features (ACF) for pedestrian detection, improving the speed without sacrificing performance, approximating features on a finely sampled pyramid. Felzenszwalb et al. [41] developed a method for object detection, based on mixtures of multiscale deformable models, using discriminative training of classifiers that make use of latent information.

Currently, the SOTA methods rely on deep learning, mostly on Convolutional Neural Networks (CNNs). These methods improved the performance of the object detection problem considerably [1,2,30,42]. Many of the generic object detection techniques were used as a base for modern pedestrian detection methods. One of the first CNN-based methods was proposed by Angelova et al. [43]. Cascade classifiers and deep neural network features were used, resulting in a fast and accurate method, that runs in real-time on the Caltech Pedestrian detection benchmark.

Cai et al. [44] developed a boosting algorithm called CompACT, by using a cascade design, optimizing a risk that accounts for both accuracy and complexity, and enabling the use of features with different complexities in a single detector. This includes a cascade combining CNNs with an object proposal mechanism, thus obtaining good results on Caltech and KITTI benchmarks. Hosang et al. [45] performed several experiments with different CNN architectures available at that time, avoiding custom designs adapted for pedestrian detection. The authors show competitive results on Caltech and KITTI benchmark datasets. Zhang et al. [33] proposed a method based on the Region Proposal Network (RPN) followed by cascaded boosted forests to classify the region proposals. Thus, features of arbitrary resolutions from any layers are combined, and hard negative mining is performed, overcoming the limitations of the original Faster R-CNN method. Brazil et al. [10] proposed a multi-task infusion framework for joint semantic segmentation and pedestrian detection, obtaining SOTA results on the Caltech dataset, and competitive performance on the KITTI dataset.

Zhou and Yuan [11] developed a method for both pedestrian detection, and occlusion estimation, using a CNN with two branches. The first branch was for full body estimation, and the second was for visible body part estimation. Both branches produce outputs that complement each other, improving detection performance. The method was assessed on the Caltech and CityPersons datasets, obtaining excellent results in detecting both non-occluded and occluded pedestrians.

Liu et al. [12] proposed a Single Stage Detector (SSD) method, named Asymptotic Localization Fitting (ALF), which stacks a series of predictors to evolve the default anchor boxes of SSD, step by step, closer to labeled boxes, and then uses a pedestrian detection architecture called ALFNet. This method improved accuracy while maintaining the efficiency of single-stage detectors, achieving SOTA performance on the CityPersons and Caltech datasets.

Liu et al. [13] proposed the CSP method, in which the pedestrian detection task is considered to be a high-level semantic feature detection, predicting the pedestrian center and scale using CNNs. This simple method reached competitive results in both detection and computing times on several pedestrian detection benchmarks.

Yin et al. [46] proposed a method called DA-Net, utilizing a two-stage detector Feature Pyramid Network (FPN) and incorporating a Dense Connected Block (DCB), which comprises a Channel-Wise Attention Module (CWAM) and a Global Attention Module (GAM). By adding several DCBs to the network, the prediction layers captured richer semantic information from targets, leading to more precise target localization. The method was assessed on CityPersons and one of the evaluations was performed on the Heavy subset, obtaining good results.

Lin et al. [47] proposed a pedestrian detector called PedJointNet, that simultaneously regresses two bounding boxes to the head–shoulder and full body regions based on a feasible object detection backbone. The detector achieved excellent performance in detecting both non-occluded and occluded pedestrians. The method was assessed on the CUHK-SYSU, TownCentre, and CityPersons datasets.

Cai et al. [48] proposed an anchor-free and proposal-free pedestrian detector, called Pedestrian-as-Points Network (PP-Net), which finds a better trade-off between accuracy and efficiency. The authors modeled pedestrians as single points, i.e., the center point of the instance, and then predicted the pedestrian scale at each detected center point. To avoid the high-level information loss on the top-down pathway, a Deep Guidance Module (DGM) was built at the top of the backbone. They obtained SOTA results on Caltech and CityPersons benchmarks.

Zhang et al. [49] assessed the performance of a Faster R-CNN pedestrian detector using a new dataset, CityPersons, that consists of person annotations on the Cityscapes dataset. The diversity of this dataset allowed training of a single model that generalizes well over various benchmarks.

Li et al. [50] developed a pedestrian detector based on YOLOv7, aimed to improve the detection of obscured pedestrians. For this purpose, the default backbone for YOLOv7 was replaced with a lightweight MobileNetV3 backbone. Then, a high-resolution feature pyramid structure was used to improve missed detection of hidden pedestrians, and an attention mechanism was used to lower the redundant bounding boxes. The method was applied to the CrowdHuman dataset, obtaining promising results.

Liu et al. [51] developed a pedestrian detector in a foggy traffic environment, named YOLO-GW, using the dark channel de-fogging algorithm, in conjunction with a YOLOv7 detector. Also, an ECA module and a detection head were added, aimed to improve object classification and regression. Results showed an improvement in the frame rate by 63.08%, and detection using mAP metric increased by 9.06%.

Braun et al. [52] assessed the generalization capacity of four deep learning object detectors applied to pedestrian detection: Faster R-CNN, R-FCN, SSD, and YOLOv3, using a new dataset, EuroCity Persons. They studied the effect on the detector performance for many variables related to training set size, dataset diversity and detail, and annotation quality. It was observed that pre-training with very large sets outperforms using only target training sets.

Chao et al. [53] also evaluated the generalization of human detectors. For this purpose, they created a large dataset, CrowdHuman, and assessed the cross-dataset generalization, obtaining new SOTA results on the Caltech, CityPersons, and Brainwash datasets. They demonstrated that the proposed new dataset could serve for pre-training human detectors.

Hasan et al. [20] performed extensive assessment using many SOTA pedestrian detection methods. They tested their domain generalization capacities on certain popular general object detection methods, not specifically designed for pedestrian detection. The authors found that these general methods performed better compared to specific pedestrian detectors when cross-dataset experiments were performed. In general, the methods reached good detection performance when trained and tested on the same dataset, but results worsened when assessed on a different dataset.

The triplet loss function has been used in different machine learning and computer vision tasks, beginning with the work of Schroff et al. [3], that used this function in the context of face recognition, proposing a method named FaceNet, that obtained a new record for accuracy on the LFW and YouTube Faces datasets. Other studies in face recognition using triplet loss were developed by Parkhi et al. [54], Trigueros et al. [55], Boutros et al. [56], Yeung et al. [57], and Feng et al. [58].

Triplet loss has also been used successfully in the context of person re-identification in recent years. This problem is related to pedestrian detection, but presents some important differences, and is defined as the task of identifying and matching the same individuals either across various cameras or across time within a single camera [59]. Along this line, many methods for person re-identification using triplet loss and reaching SOTA results have been proposed in the past few years, as in [60,61,62,63,64,65]. Interesting work was proposed by Wang et al. [66], in which the triplet loss function is used to adjust the feature distance of each pedestrian to distinguish different pedestrians in crowded scenarios. However, in our method, we use the triplet loss function to help cluster together the features of all the pedestrians, instead of identifying single individuals.

The triplet loss function has been successfully used for domain generalization in various tasks. The research of Lee [67], applied the triplet loss function for cross-corpus speech emotion recognition to generalize across domains. Yu et al. [68] introduced an adapted triplet loss as a novel approach to mitigate bias in triplet selection, and address distribution shift in selected triplets, evaluating different image classification datasets. Wang et al. [69] introduced a novel domain generalization framework, EISNet, which learns to generalize across diverse domains concurrently by utilizing both extrinsic relationship supervision and intrinsic self-supervision, particularly for images from multiple source domains. In the work of Dou et al. [70], they used a model-agnostic learning paradigm to expose the optimization to domain shift, introducing two complementary losses that regularize the semantic structure of the feature space explicitly. Deng et al. [71] studied metric learning within domain adaptation, introducing a similarity-guided constraint in the form of a triplet loss, where each triplet is taken from both the source and target domains.

Finally, we can mention other machine learning tasks, not restricted to computer vision, where triplet loss has been used. Here we can cite some tasks such as object tracking [72,73,74], speaker recognition [75,76], intention detection for spoken language understanding in dialogue systems [77], remote sensing image retrieval [78], 3D gesture recognition [79], automatic music cover detection [80], and low-light image enhancement [81].

Currently, there are many benchmarks for assessing pedestrian detection; for example, Daimler [82], INRIA [39], ETH [83], TUDBrussels [84], and WiderPedestrian [85]. Many of these datasets were captured from surveillance scenarios, and were not suitable for autonomous driving applications. Instead, there are other datasets specifically aimed at autonomous driving, such as Caltech [21], KITTI [86], CityPersons [49], and EuroCity Persons [52].

From our literature review, it can be concluded that many methods reached good pedestrian detection performance when trained and tested on the same datasets. However, results worsened significantly when cross-dataset experiments were performed using a testing dataset different from that used for training. The ability to perform well on unseen scenarios is crucial for methods to be deployed in real-life applications. For example, the pedestrian detector in an autonomous vehicle is effectively a cross-dataset scenario, in which most of the data seen is new for the detector. We concluded that cross-dataset testing is a problem that has not yet been solved in the current SOTA. Another important issue present in our literature review is the limited ability to detect pedestrians with a high degree of occlusion. This can be confirmed by analyzing the results for the heavy partition on the CityPersons dataset, whose current SOTA results are still far from the results obtained for the reasonable partition, an easier scenario on which methods are able to obtain useful results for real-life applications.

## 3. Materials and Methods

Most two-stage pedestrian detectors that are present in the literature have two losses in the second stage: one loss for bounding box regression, and another for classification. Two-stage methods are preferred because they have achieved better performance, sacrificing speed when compared to the one-stage approaches. The two-stage methods proved to be successful if the train and test datasets come from the same domain, i.e., belong to the same dataset, but when these methods are evaluated on new, unseen datasets, performance drops significantly, as shown by Hasan et al. in [20]. To overcome this issue, we designed a new head in the second stage, to try concentrating pedestrian examples in the feature space explicitly, independent of from which dataset they came, and separating the background examples from them. Our proposed method uses the concept of triplets that has been used successfully in other computer vision tasks [3,58]. We used the triplet loss function, which involves three samples in the calculation: an anchor sample, a sample of the same class as the anchor, and a sample with a different label. In this way, the network learns to minimize the distance between samples of the same label, while maximizing the distance of samples of different labels.

### 3.1. Two-Stage Object Detectors

In this work, we targeted our effort at improving the two-stage detector architecture. We performed experiments using Faster R-CNN [2], and Cascade R-CNN [32], which both belong to the R-CNN methods family. Cascade R-CNN outperforms Faster R-CNN in many object detection benchmarks, but we also included the latter method because it is still used, as is reported in the literature, to serve as a baseline for comparison. Both methods approach the detection as a multi-task learning problem, combining classification and bounding box regression.

Faster R-CNN [2] is based on Fast R-CNN [1], but the main difference includes a region proposal stage that is performed using a novel Region Proposal Network (RPN) that shares convolutional features with the detection network, thus enabling nearly cost-free region proposals. This RPN is a fully convolutional network that predicts bounding boxes and objectness scores at each position, at the same time. This improvement was developed to reduce the execution time of the region proposal stage which acted as a bottleneck in terms of speed.

Cascade R-CNN [32] tackles the problem of noisy detections when a detector is trained with a low IoU threshold, such as 0.5 in most cases. This is not trivial to solve, since performance tends to degrade when the IoU thresholds are increased. To overcome this problem, a sequence of detection heads is trained with increasing IoU thresholds, stage by stage, to be more selective sequentially against close false positives. In this manner, false positive anchors are filtered out, generating better-quality proposals.

**Figure 1:**
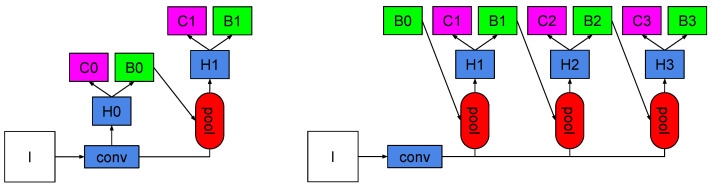
Generic architectures for Faster R-CNN (**Left**), and Cascade R-CNN (**Right**). I is the input image; conv is the convolutional backbone; pool is the region-wise feature extraction; H represents the network heads where C is a classification head, and B is a bounding box regression. B0 represents proposals for both architectures.

The generic architectures of both Faster R-CNN and Cascade R-CNN are shown in Figure 1. In Faster R-CNN, the first stage is a region proposal sub-network H0, which operates on the entire image, generating preliminary detection hypotheses, known as object proposals, and denoted as B0. In the second stage, hypotheses are processed by a region-of-interest detection sub-network, H1, denoted as the detection head, assigning a final classification score, C1, and a refined bounding box, B1. This is analogous to Cascade R-CNN.

### 3.2. Triplet Loss

**Figure 2:**
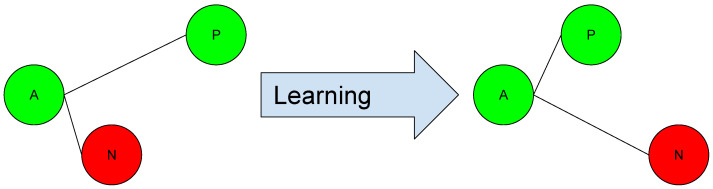
Triplet loss. The network learns to minimize the distance between samples of the same label while maximizing the distance between samples of different labels.

The triplet loss function trains the neural network to embed features of the same class, while maximizing the distance among embeddings of different classes. An anchor is chosen, along with one negative and one positive sample, to compute the loss. As a result, the triplet loss maximizes the inter-class distance explicitly while it minimizes the intra-class distance, where a margin term is used to determine the decision boundary between positive and negative pairs. Generally, this family of functions is applied to the sample projection (Embeddings), performed by a neural network. The behavior of this type of function is shown in Figure 2. This function has been used successfully in many machine learning and computer vision applications, for example, in face recognition [3,54,56], person re-identification [59,62,63], object tracking [72,73,74], and speaker recognition [75,76], etc.

Formally, this function is defined by triplets of embeddings, defining the following concepts:

- An anchor sample *a*, for example, a pedestrian.
- A positive sample *p*, with the same class as the anchor.
- A negative sample *n* of a different class, for example, background.

For some distance *d* on the embedding space (typically Euclidean distance), the loss for a triplet (a,p,n) is defined as:

(1) L=max(d(a,p)−d(a,n)+margin,0)

Our goal is to minimize this loss function, pushing d(a,p) to 0 and d(a,n) to be greater than d(a,p)+margin. As soon as *n* becomes an “easy negative”, the loss becomes zero.

Based on the definition given in Equation (1), there are three categories of triplets:

- Easy triplets: Triplets which have a loss of 0, because d(a,p)+margin<d(a,n);
- Hard triplets: Triplets where the negative is closer to the anchor than the positive, i.e., d(a,n)<d(a,p);
- Semi-hard triplets: Triplets in which the negative sample is not closer to the anchor than the positive sample, but still have positive loss: d(a,p)<d(a,n)<d(a,p)+margin.

**Figure 3:**
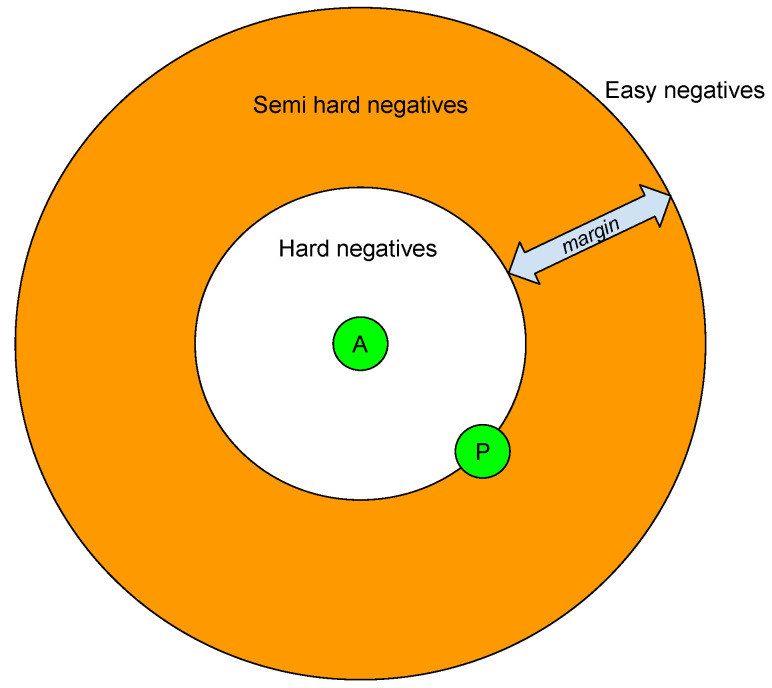
Categorization of negative samples according to the relative distances with positives (P) and anchor (A).

According to the previous definition, we can categorize the negative samples into hard negatives, semi-hard negatives, and easy negatives. This is related to the location of the negative sample in relation to the anchor and positive samples within the embedded space. It can be observed in Figure 3.

The strategy for triplet selection is a crucial step for achieving good detection performance, as stated in the literature [65,72]. It is necessary to select which triplets the network will process, because if all possible triplets were generated, many of them would be “easy” triplets, and would not contribute to the training. This would result in slower convergence, as all the triplets require computation through the network. Therefore, it is crucial to select active triplets, i.e., hard and semi-hard triplets, that can contribute to improving the model during training [3]. In our case, we followed the strategy used in [3], using online negative exemplar mining, that ensures increasing difficulty of triplets as the network training progresses. For this purpose, we generated the triplets online, selecting the hard positive/negative exemplars from within a mini-batch. It is also important to avoid the problem of unstable training. For this purpose, a meaningful representation of the anchor-positive distances needs to be guaranteed. It is necessary to ensure that a minimal number of exemplars of each class is present in each mini-batch. We adapted the procedure used in [3] to our particular case. We ensured that for each image, during training, there is at least one pedestrian that generates a few positive samples, after the ROI pooling stage, that are used for generating the triplets.

### 3.3. Modified Classification Head with Embeddings

The proposed method focuses on the classification head that occurs immediately after the pooling feature extraction stage, with the features calculated from the regions generated by the RPN. As stated previously, current two-stage object detectors are designed for multi-task learning, performing classification and bounding box regression simultaneously. We modified the current final classification head, adding a third loss function, so that the distances among the embeddings are optimized according to the triplet loss function defined previously.

The behavior of a regular two-stage object detector can be summarized as follows: First, an image is taken, which generates multiple regions of interest (ROIs) that are fed into a fully convolutional network. Then, each region is pooled into a fixed-size feature map and projected onto a feature vector by a fully connected network. These vectors enter a network with two sibling output layers, on which the first delivers a discrete probability distribution $p=\left(p_{0},\ldots ,p_{K}\right)$ for each ROI over the K+1 categories, and the other provides regression offsets $t^{k}=\left(t_{x}^{k},t_{y}^{k},t_{w}^{k},t_{h}^{k}\right)$ for bounding boxes for each of the *K* classes. Each ROI is labeled with a ground truth *u* and an annotated bounding box *v*. For training, a multi-task loss function was used, given by the following equation:

(2) $$L\left(p,u,t^{u},v\right)=L_{\mathrm{cls}}\left(p,u\right)+\lambda \left[u\geq 1\right]L_{\mathrm{loc}}\left(t^{u},v\right)$$

with the classification function $L_{\mathrm{cls}}\left(p,u\right)=-\log\left(p_{u}\right)$ defined as the log loss for true class *u*, while the regression function $L_{\mathrm{loc}}$ is usually the Smooth $L_{1}$ function, this function being a robust $L_{1}$ loss that is less sensitive to outliers than the $L_{2}$ loss. The weight for balancing both functions is represented by λ. It can be observed from the above equation, that there is no notion of a ground-truth bounding box for background ROIs, so $L_{\mathrm{loc}}$ is neglected [1].

**Figure 4:**
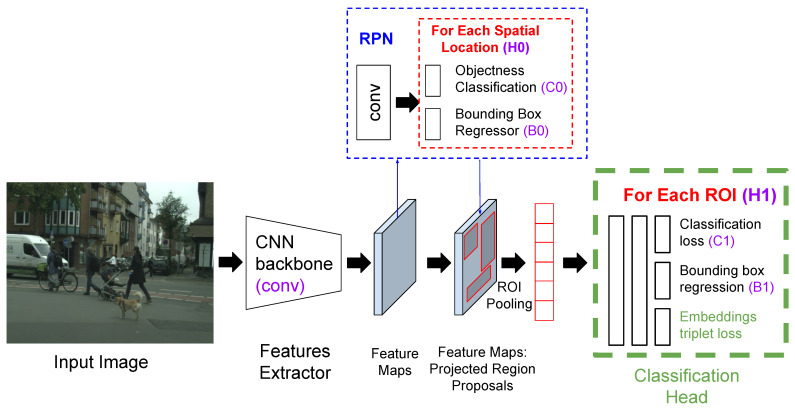
Faster R-CNN architecture with the proposed modified head. Our contributions are shown in green, with the addition of the triplet loss function in the classification head. In purple are the blocks corresponding to Figure 1.

In our work, the samples are the ROIs projected onto the embedded space, generated by the ROI extractor. They can therefore be compared since the vectors have the same length. The proposed architecture, but in a Faster R-CNN framework, can be observed in Figure 4. We applied the triplet loss function using these features to emulate somewhat the behavior of an embedded space in, for example, a face recognition task, where faces of the same subject must be closer, concentrated in space, and farther away from other subject faces. In this case, we want the features for the pedestrians to lie closer than the features for the background.

The loss function for the new classification head is defined as follows:

(3) $$L\left(p,u,t^{u},v,f\right)=L_{\mathrm{cls}}\left(p,u\right)+\lambda_{1}\left[u\geq 1\right]L_{\mathrm{loc}}\left(t^{u},v\right)+\lambda_{2}L_{\mathrm{emb}}\left(f,u\right).$$

In the above equation, $L_{\mathrm{cls}}$ represents the classification loss function, and $L_{\mathrm{loc}}$ represents the regression loss function. Finally, $L_{\mathrm{emb}}$ represents the function applied to the embeddings; in this case, the Triplet Loss, which operates on the extracted features *f* after the region of interest extraction. On the other hand, $\lambda_{1}$ represents the weight assigned to the regression function, and $\lambda_{2}$ represents the weight assigned to the function applied to the embeddings.

For Faster R-CNN, the head was modified by adding triplet loss directly as a third loss function, complementing the classification and bounding box regression losses. For Cascade R-CNN, as observed at the right in Figure 1, we can use this new modified head to replace any of the existing three heads, H1, H2, or H3. Several experiments were performed to find the optimal location of this new head in the Cascade R-CNN approach. In all the experiments performed, the margin used was 1.0, as a default value, and it performed well. We used this value because it was used in several deep learning frameworks [87,88].

### 3.4. Experiments

For training, we used the same progressive pipeline protocol described in [20], which allowed us to have an advantage in enhancing pedestrian detection performance over datasets obtained from multiple sources. This pipeline trains detectors using a dataset that is diverse, but different from that of the target domain. Subsequently, the pipeline fine tunes the detectors on a dataset that closely resembles the target domain. As expected, we used only the training subset of each dataset for training and the evaluation subset for the performance assessment.

We used CityPersons for training Faster R-CNN and Cascade R-CNN with triplet loss, in the same domain experiments as follows: We used the training partition available in the CityPersons dataset for training. For the cross-dataset experiments, we used the validation partition of CityPersons for evaluation; we did not use the training partition. We used the validation partition instead of the testing partition, because the latter is intended as a challenge dataset, with no available annotations, compared to the validation partition, which does have annotations available. This is the standard procedure used in the literature [13,14,20,89]. About the data augmentation, we used only vertical flipping. In future work, we can apply other data augmentation operations, aimed at improving the generalization capabilities of our method.

We performed experiments on different detectors comparing their performance. As detectors, we used Faster R-CNN [2] and Cascade R-CNN [32]. The backbone used was HRNet [90], because in the SOTA this backbone has shown performance advantages over other backbones, such as ResNet50 or ResNeXt [20].

For assessing the detector performance, we used the standard protocol available whose use has been reported extensively in the literature [49,52,91]. The $MR^{-2}$ metric, also called MR, is defined as the log average miss rate over the False Positive Per Image (FPPI), computed by averaging the miss rate, at nine FPPI rates evenly spaced in log-space, in the interval $\left[10^{-2},10^{0}\right]$. This metric was chosen because it allowed us to compare the results obtained by our method with the current SOTA. It must also be mentioned that this metric was used in the articles that introduced the most used datasets in the field, Caltech [91] and CityPersons [49]. It was presented as an evaluation metric for pedestrian detection by Dollar et al. [91], in which the Caltech dataset was introduced. This metric serves as a suitable indicator for algorithms applied in real-world applications. As this metric quantifies the error, a lower value represents a better performance for the assessed algorithm. This metric has been used extensively in recent years for the pedestrian detection problem, as stated in [12,13,14,20,89,92]. The methods were assessed at different pedestrian sizes and occlusion levels, as defined on Table 1.

### 3.5. Datasets

The datasets used in our work are the following:

- CityPersons [49]: This dataset is a subset of the Cityscapes dataset [93], but with only person annotations. The images were captured in different cities of Germany, and in adjacent countries. There are 2975 images for training, 500 for validation, and 1575 for testing. There is a 6.47 average number of pedestrians per image. Annotations are provided for a person’s visible region and full body. To be able to compare our results to those of the SOTA, we used only the train and validation subsets in this work.
- EuroCity Persons [52]: This is a large-scale dataset recorded in 31 European cities, with a variety of different scenarios. According to the time of recording, EuroCity Persons provides two subsets: daytime and nighttime. There are 21,975 images for training, with an average of 9.2 pedestrians per image. To be able to compare our results to those of SOTA we used only the daytime training subset.
- WiderPedestrian [85]: This dataset addresses the problem of pedestrian detection in unconstrained environments. The images were acquired in autonomous driving and surveillance applications. The dataset contains 90,000 images for training, with an average of 3.2 pedestrians per image. We also used only the training subset in this case to be able to compare it to other SOTA results.

**Figure 5:**
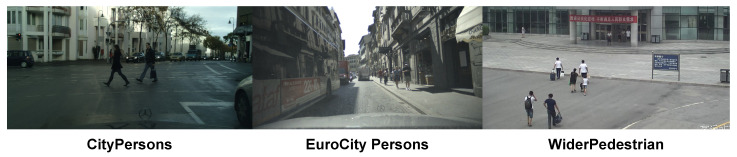
Examples of images from the datasets used in this work.

Examples of the datasets employed in this work are shown in Figure 5.

We focused our work on obtaining the best pedestrian detection results on the validation partition of the CityPersons dataset, and compared them to those of the SOTA.

All the experiments were conducted using the MMDetection [94] open source object detection toolbox, which is based on PyTorch, as a part of the OpenMMLab project. The GPU used for the experiment was an NVIDIA GeForce 2080TI with 11GB of RAM. Similarly, as in other SOTA work, we used only vertical flipping for data augmentation [20]. The optimizer used was SGD, with learning rate = 0.002, momentum = 0.9, and weight decay = 0.0001.

## 4. Results and Discussion

In this section, we performed several experiments applying our newly developed Classification Head with Triplet Loss to Faster R-CNN and Cascade R-CNN detectors. Then, we performed an ablation study to assess the impact of our head compared to the regular detectors. Finally, we performed a comparison study with the SOTA, and reviewed some qualitative results, comparing our method with a SOTA method.

### 4.1. Faster R-CNN with Triplet Loss Head Results

The first set of experiments was performed using our new proposed head, with the triplet loss function, replacing the standard classification head of a Faster R-CNN object detector with an ImageNet pre-trained HRNet backbone. The results obtained are shown in Table 2.

We can see in Table 2 that a small contribution from the triplet loss function to the classification head obtains the best results. Conversely, increasing the weight for triplet loss leads to a significant decrease in performance.

Cross-dataset generalization experiments were then performed. We trained a Faster R-CNN detector using WiderPedestrian, and evaluated it on CityPersons, as can be seen in Table 3. From the results shown in Table 2, we can observe that using weights in the interval [0,0.25] resulted in the lowest error. Therefore, we performed cross-dataset generalization experiments, focusing only on low values, in the range [0,0.25], for the triplet loss weight.

The results shown in Table 3, are worse than those shown in Table 2 because the source dataset in Table 3 is different from the target dataset. This effect is amplified on the heavy subset, the most difficult one on the benchmark. However, in Table 2, the source and target datasets are the same. If we observe the results in Table 3, the performance using triplet loss is the best, in general terms for all the subsets, when the weight is 0.2. Nevertheless, in the following experiments, the results improved significantly when the model was fine-tuned using the triplet loss on datasets that are closer to the target domain, in this case, EuroCity Persons.

### 4.2. Cascade R-CNN with Triplet Loss Head Results

The next step was to perform the experiments using the Cascade R-CNN framework. There are multiple classification heads in this detector, and, therefore, we had to choose the place to apply the triplet loss function. For this purpose, we performed several experiments, modifying the head where triplet loss is applied, and the weight for the triplet loss function within the selected head. A summary of the experiments performed is shown in Table 4, where, in general terms, applying triplet loss in the first classification head seems to perform better, since it performs well on the reasonable, small, and heavy subsets at the same time. The detection performance increased compared to the results obtained using Faster R-CNN as shown in Table 2. In this case, the best result for the reasonable subset was obtained using a small weight of 0.05 for the triplet loss function in the first classification head.

Proceeding in a similar fashion to Faster R-CNN, we performed cross-dataset experiments for Cascade R-CNN. In this case, we trained our models on WiderPedestrian, and then evaluated them on CityPersons. Since the results seemed to be better rounded for all the datasets in which we applied our head in the first head, we chose this head for the following experiments. We also focused on small values for the weight, because, as stated previously, it seems that only a small weight has the most positive impact on the detector. Results for this set of experiments can be observed on Table 5.

The same effect that occurred with Faster R-CNN can be seen here, where the results shown in Table 5 are worse than those shown in Table 4. This is also because the source dataset on Table 5 is different from the target dataset. Again, this effect is most noticeable in the heavy subset. The best global result for Cascade R-CNN was obtained by applying triplet loss with a weight of 0.15 to the first classification head, H1. This weight worked well in all the subsets on CityPersons.

### 4.3. Ablation Study

In this section, we compare the best results for cases of Faster R-CNN and Cascade R-CNN with Triplet Loss, with our new head, and without it.

In Table 6, results for Faster R-CNN and Cascade R-CNN detectors, trained and evaluated on CityPersons, when using our modified head vs. the regular detector are shown.

We can observe in Table 6, that for both Faster R-CNN and Cascade R-CNN, a small contribution from the triplet loss function to the classification head improves the performance on almost all the subsets in CityPersons. For the Faster R-CNN method, results in the reasonable subset improved moderately, but the improvement was significant on the heavy subset. For Cascade R-CNN, results on the reasonable subset improved significantly, and moderately on heavy and small subsets.

Then, in Table 7, results are shown for Faster R-CNN and Cascade R-CNN detectors, trained on WiderPedestrian and evaluated on CityPersons, when using our modified head vs. the regular detector.

We can see in Table 7 that using triplet loss again improved the results compared to the results obtained without it. This behavior can be seen for both Faster R-CNN and Cascade R-CNN. The improvement in the performance is especially evident for the Cascade R-CNN, where the error for the reasonable subset decreased by 2.2%, 3.5% for the small subset, and an impressive 6.5% for the heavy subset. This shows the impact of the triplet loss head on a cross-dataset scenario for the most difficult samples in the dataset, that belong in the heavy subset.

### 4.4. Comparison Study

SOTA results, for the CityPersons benchmark, can be observed in Table 8. All these results were obtained using the target dataset for training and testing, and therefore, no generalization capabilities were tested.

It can be seen that when comparing the best Faster R-CNN results on Table 8 (15.4% on the reasonable subset), with the best result on Table 2 (13.7% on the reasonable subset), we observe a significant reduction in $MR^{-2}$ of about 1.7% for the reasonable subset. Also, the results on the small subset improved by 7.3%, from 25.6% when using Faster R-CNN with a VGG16 backbone and without triplet loss, to 18.3% using triplet loss and an HRNet backbone. This is a combined effect of the triplet loss function, and the HRNet backbone, because in [49], an older VGG16 [95] backbone was used.

A comparison including the best results of our method, using Faster R-CNN and Cascade R-CNN detectors, can be seen in Table 9, compared to those of the SOTA for domain generalization using WiderPedestrian, taken from Hasan et al. [20]. It can be observed in the third row of Table 9, that the Faster R-CNN method with triplet loss outperforms the Cascade R-CNN algorithm without triplet loss shown in the first row, the latter being a modern method that on most object detection tasks outperforms Faster R-CNN [20,32]. The fourth row of Table 9 shows the results of our method using Cascade R-CNN. It can be observed that our results outperform those of the regular Cascade R-CNN shown on the first row, obtaining an improvement for the reasonable subset by 2.2%, and 6.5% on the heavy subset.

Finally, we performed a set of experiments to assess the generalization capabilities of our method, applying Hasan’s progressive training pipeline [20], and always using the CityPersons benchmark as a target set to be able to compare our results to those of the SOTA. The best results obtained for this set of experiments are shown in Table 10. We followed the same sequence of cascade training as in the SOTA to be able to compare our results to those already published. We performed the first training on the WiderPedestrian dataset, and then, with the best result obtained (last row of Table 9), we fine-tuned using the EuroCity Persons daytime subset. We obtained the best result with a weight of 0.1 for the first classification head (H1) in the Cascade R-CNN detector. In summary, we trained a detector on WiderPedestrian, using a weight of 0.15 on H1, and then, fine-tuned it on EuroCity Persons, using a weight of 0.1 on the H1 head. As shown in Table 10, our results outperform the current SOTA on two of the three subsets of CityPersons, i.e., the small and heavy subsets, and obtained a comparable performance on the reasonable subset. The difference in performance is about 1.5% better for the heavy subset, which has been shown to be the most difficult subset in CityPersons in the literature [13,20,47,48]. We hypothesize that the results in the target subset will improve with our method when using additional datasets in the training pipeline, since the base performance is greater.

### 4.5. Qualitative Results Comparison and Discussion

**Figure 6:**
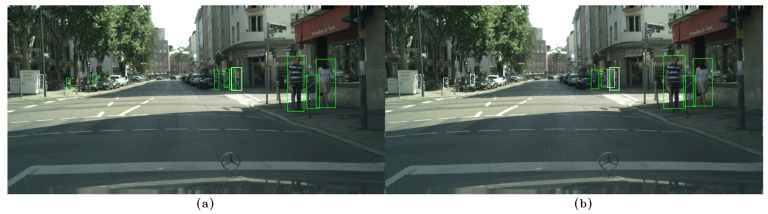
(**a**) Results on the CityPersons dataset using our method. (**b**) Results on the CityPersons dataset using CSP [13]. False negatives are shown in white and true positives in green. In (**a**), occluded false negatives can be observed: At the right of the image, a missed detection (in white) is generated by another pedestrian. At the left, a short pedestrian is missed because of a high degree of occlusion caused by an object. Also, the method performs well on short pedestrians, as is shown in the left portion of the image. We can also observe that our method obtains better results compared to CSP, shown in (**b**), especially on the pedestrians present in the left portion of the image, where CSP is unable to detect any pedestrians, compared to using our method, with which we can detect 2 out of 3 short pedestrians.

**Figure 7:**
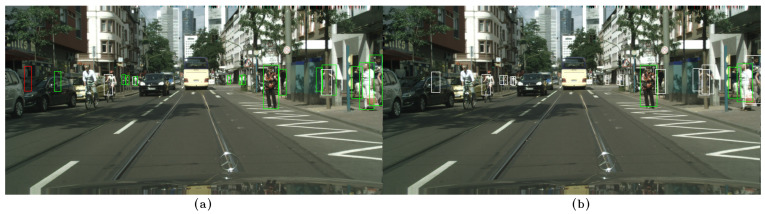
(**a**) Results on the CityPersons dataset using our method. (**b**) Results on the CityPersons dataset using CSP [13]. False negatives are shown in white, false positives in red, and true positives in green. In (**a**), at the left of the image, two missed detections (in white) and a false positive (in red) can be observed, but the false positive is effectively a pedestrian. On the right, two missed detections are observed. This example shows good performance on pedestrians of both average and short height. We can also observe that our method, obtains better performance on the left portion of the image, compared to CSP, shown in (**b**), since CSP does not detect pedestrians, who are of short stature. Also, in the right portion of the image, several false negatives (in white) are shown, compared to using our method, which detects most of the pedestrians.

**Figure 8:**
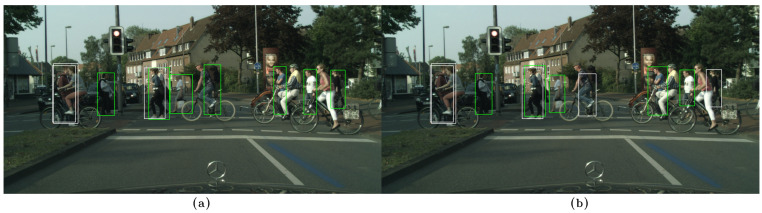
(**a**) Results on the CityPersons dataset using our method. (**b**) Results on the CityPersons dataset using CSP [13]. False negatives are shown in white and true positives in green. In our method, two false negatives (in white) that were generated by another pedestrian are shown in the left part of the image. There is also another false negative (in white) in the middle, also caused by another pedestrian. It should be noted that the cyclists are ignored intentionally in the annotations, because they do not belong to the pedestrian class. The CSP method, shown in (**b**), generates two additional false negatives (in white) in the middle and at the right of the image.

**Figure 9:**
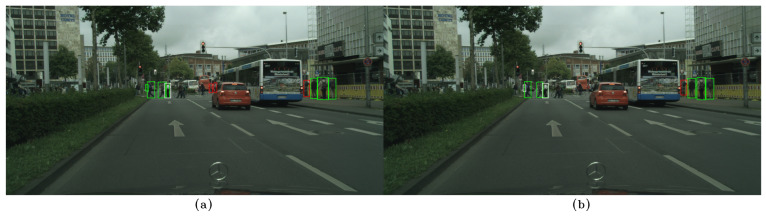
(**a**) Results on the CityPersons dataset using our method. (**b**) Results on the CityPersons dataset using CSP [13]. False negatives are shown in white, false positives in red, and true positives in green. In (**a**) a false negative caused by another pedestrian is observed (in white), in the middle. Also, in the middle, and at the right, red boxes are observed that are reported as false positives (in red), but if we observe carefully, they are pedestrians that are not annotated in the CityPersons dataset. The results of the CSP method are shown in (**b**). We can see a short pedestrian missed in the middle, and the two short pedestrians that our method detects but that are not annotated, are missed by CSP.

**Figure 10:**
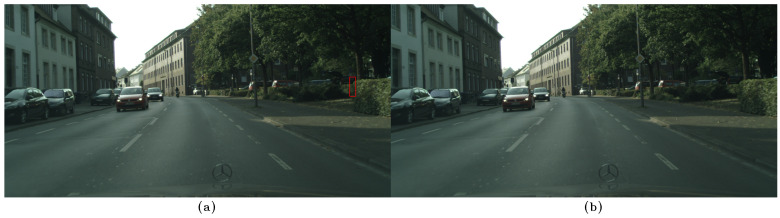
(**a**) Results on the CityPersons dataset using our method. (**b**) Results on the CityPersons dataset using CSP [13]. In (**a**) our method shows a false positive on the right side (in red), but if we observe carefully, there is a pedestrian that is not annotated in the CityPersons dataset. This pedestrian is not detected by CSP as shown in (**b**).

In the following examples, qualitative results of our model on the CityPersons dataset can be observed and compared to those of CSP [13], where the images on the left, (a), show the results of our method, while the images on the right, (b), show the results of CSP. False negatives are shown in white, false positives in red, and true positives in green. In Figure 6a, Figure 7a, and Figure 8a, some false negatives are shown when observing the results obtained by our method, but under careful observation, it can be seen that most of the missed detections occur in areas where occlusion is generated by another pedestrian. In cases of other occlusion types, e.g., generated by cars or other objects, the method performed well, even with pedestrians of low visibility and short stature. Specifically, in Figure 6 and Figure 7, a significant difference in performance can be observed between both methods, with our method able to detect more of the most difficult pedestrians when compared to CSP, especially those who are short and occluded. In Figure 8a, our method shows better results when applied to occluded pedestrians. In general terms, our method shows some errors that are mainly caused by occlusions made by other pedestrians, and it shows that it handles occlusions better than CSP in Figure 8b. Performing a qualitative analysis of the whole testing set, this seems to be the main cause of our method missing occluded pedestrians. It must be noted that riders (cyclists and motorists) are intentionally not detected, since they belong to another class in the Cityscapes dataset. In Figure 7a, a false positive can also be noted in red, but when observed carefully, it seems to be a highly occluded pedestrian that is not annotated in that location. Figure 9a shows instances of many false positives, and also one false negative, that seems to be caused by another pedestrian, as in Figure 6a, Figure 7a, and Figure 8a. Observing carefully, it can be seen that all the red bounding boxes are effectively pedestrians, some of which are highly occluded, but they are counted as false positives according to the evaluation procedure in the literature. Comparing our results to those of CSP, Figure 9b shows only one of these misses labeled false positives, resulting in worse performance (also not detecting a short pedestrian in the middle of the scene). Finally, in Figure 10a, a single detection can be observed, in red, that is also a highly occluded pedestrian. This pedestrian is not detected by CSP, as can be seen in Figure 10b.

From the qualitative results obtained, it can be observed that the proposed method works well on difficult images, handling occlusions and scales better than CSP [13], and even detecting some difficult pedestrians, i.e., those that in the evaluation are marked as false positives (red bounding boxes), but are effectively pedestrians, some of whom are highly occluded. Our method is able to manage pedestrians in different scales, with different illumination and occlusion degrees, and even avoid detecting bicycle riders, who are visually highly similar to pedestrians, creating an additional degree of difficulty. These results could be explained by the triplet loss design that tries to explicitly cluster all types of pedestrians together, independent of their degrees of occlusion or size. Therefore, the network learns that different degrees of occlusion and scale must be close in the feature space. Several examples of good detections of highly occluded pedestrians are shown in Figure 6, Figure 7, Figure 8, Figure 9 and Figure 10. One limitation that can be observed occurs when occlusions are generated by other pedestrians, instead of by fixed objects in the scene, for example, cars or trees. Future research could be focused on how to handle this type of occlusion.

The results obtained also showed that our method can generalize well from datasets with different scenarios. The dataset on which our method is evaluated, CityPersons, has images captured in different cities in Germany and its neighboring countries, during three seasons and under various weather conditions. On the other hand, training data comes from WiderPedestrian and EuroCity Persons, with WiderPedestrian being composed of surveillance and car-driving images, with very different camera angles, object scale, and illumination, and even some images captured at night. EuroCity Persons was captured in 31 cities of 12 European countries, spanning a large geographical area, during four seasons, which implies a variety of clothing styles, i.e., light/short for summer and thick/long for winter, and weather conditions being dry or wet. Having all these different conditions in the training set, and the triplet loss capability to cluster together samples of the same class, in this case, pedestrians, allows our detector to generalize well, because it can project samples closer together in the feature space, even if they are captured under different conditions, for example, in winter and summer, with rain, snow, different degrees of occlusion, etc. Even if there are fewer samples from one scenario to another, they are forced to lie together in the feature space.

The computational cost, in terms of the execution time of adding the triplet loss function to the existing classification and regression losses, is small according to our measurements. During training, the computational time increases by about 1%, with an average iteration time of 0.6853 s with the triplet loss, compared to 0.6799 s without it, while employing an NVIDIA GeForce 1080TI GPU. This could be explained by the fact that all computations performed for the new loss, require a distance computation within a mini-batch that uses up most of the time. During inference, the computational time is the same for the cases with and without triplet loss, because this loss is only used during training time. Therefore, the triplet loss does not have a negative impact while using the model for pedestrian detection, once the network is trained.

## 5. Conclusions

Pedestrian detection is one of the key tasks in computer vision for which several models have been developed in the past few years and that have shown a steady improvement over time, especially with deep-learning-based methods. Many real-world applications require high performance in pedestrian detection, such as the following: autonomous driving, robotic navigation, video surveillance, action recognition, and tracking. In this work, we developed a new pedestrian detection method using a new classification head for two-stage detectors. This method is aimed at improving the domain generalization capabilities of existing object detectors applied to pedestrians. We added a third loss function, based on the triplet loss function, to the classification and bounding box regression losses, and applied it to the embeddings generated to the regions of interest by the RPN network. This method improves the feature compactness of pedestrian samples, and therefore, features are clustered together in the feature space. We obtained SOTA results on the CityPersons benchmark, but it was done without training the method explicitly within the target dataset. We used progressive pipeline training, first using WiderPedestrian, and then fine-tuning on EuroCity Persons, achieving a major improvement on the heavy partition, which, with the current SOTA results, is the most difficult partition for the CityPersons benchmark. We obtained an $MR^{-2}$ of 9.9 for the reasonable, 11.0 for the small, and 36.2 for the heavy subsets, which surpasses the current SOTA results for the small and heavy subsets, and is highly competitive for the reasonable subset. These results showed that our method is able to generalize well in different cities and weather conditions, because of how the CityPersons dataset is composed. Also, our proposed head could be used as a new direction for improving cross-dataset performance in other pedestrian detectors with compatible architectures, or other object detection tasks, considering real-world applications such as autonomous driving and video surveillance. For future work, we think that our method would benefit from training with additional datasets in the progressive training pipeline, since the base performance of our method is significantly higher. Also, because all the images in CityPersons are captured under daylight conditions, in future work our method could be trained and tested using datasets that have nighttime images, for example, the nighttime partition of EuroCity Persons.

## Figures and Tables

**Table 1:** Different experimental settings for evaluation.

Setting	Height	Visibility
Reasonable	[50, *∞*]	[0.65, *∞*]
Reasonable small	[50, 75]	[0.65, *∞*]
Heavy	[50, *∞*]	[0.2, 0.65]
All	[20, *∞*]	[0.2, *∞*]

**Table 2:** $MR^{-2}$ results for Faster R-CNN with triplet loss, trained and evaluated on CityPersons.

Triplet Loss Weight	Reasonable	Small	Heavy	All
0.025	13.7	18.3	40.2	37.6
0.050	14.8	17.6	39.7	38.1
0.010	14.0	18.6	40.9	37.7
0.150	14.2	18.2	40.4	37.8
0.200	14.6	18.7	41.5	39.1
0.250	15.7	19.1	42.1	39.5
0.500	16.5	20.2	47.2	41.5
0.750	17.6	20.3	48.8	43.0
1.000	17.9	21.3	48.5	42.7

**Table 3:** $MR^{-2}$ generalization results for Faster R-CNN with triplet loss, trained on WiderPedestrian and evaluated on CityPersons.

Triplet Loss Weight	Reasonable	Small	Heavy	All
0.10	15.7	19.6	54.8	44.3
0.20	15.6	20.4	53.5	42.7
0.25	16.1	20.0	53.2	43.2

**Table 4:** Results for Cascade R-CNN adding triplet loss to each classification head (H1, H2, and H3 in Figure 1), trained and evaluated with CityPersons.

Head	Triplet Loss Weight	Reasonable	Small	Heavy	All
H1	0.025	13.4	17.3	38.9	36.5
H1	0.050	12.7	16.1	39.7	35.9
H1	0.100	12.8	15.5	38.8	35.8
H1	0.150	13.4	16.8	41.7	36.9
H1	0.200	13.4	16.0	40.1	36.7
H1	0.250	13.8	17.1	40.5	36.4
H1	0.500	13.9	15.5	41.8	37.1
H1	0.750	14.8	17.5	44.4	38.6
H1	1.000	14.9	19.0	44.4	38.9
H2	0.025	13.7	16.4	40.1	37.2
H2	0.050	13.7	16.1	40.7	36.4
H2	0.100	13.5	16.8	38.5	36.0
H2	0.150	13.0	16.0	39.8	35.9
H2	0.200	15.9	20.1	48.1	40.4
H2	0.250	15.3	19.6	46.5	39.3
H2	0.500	13.0	16.1	40.2	36.1
H2	0.750	16.7	22.1	50.1	41.2
H2	1.000	18.6	24.7	54.6	43.9
H3	0.025	13.5	16.6	41.5	36.7
H3	0.050	12.7	15.2	40.5	35.7
H3	0.100	13.3	14.7	40.5	36.4
H3	0.150	13.6	15.5	38.4	35.7
H3	0.200	13.5	17.4	39.6	36.5
H3	0.250	13.1	16.1	40.0	35.7
H3	0.500	13.9	16.7	38.8	36.6
H3	0.750	13.0	14.8	40.3	36.0
H3	1.000	13.2	17.2	41.3	36.3

**Table 5:** $MR^{-2}$ generalization results for Cascade R-CNN using triplet loss in the first classification head (H1 in Figure 1) trained on WiderPedestrian and evaluated on CityPersons.

Triplet Loss Weight	Reasonable	Small	Heavy	All
0.025	13.5	18.1	53.0	41.3
0.050	14.4	19.1	52.9	40.9
0.100	14.1	20.3	52.5	40.7
0.150	13.8	18.1	50.9	39.7
0.200	14.0	18.7	50.7	40.7
0.250	14.8	19.5	52.8	41.8

**Table 6:** $MR^{-2}$ results for Faster R-CNN and Cascade R-CNN, comparing the best triplet loss result against the method without it, trained and evaluated on CityPersons.

Method	Reasonable	Small	Heavy	All
Faster R-CNN (w/o Triplet Loss)	13.8	16.2	47.6	39.7
Faster R-CNN (Triplet Loss w = 0.025)	13.7	18.3	40.2	37.6
Cascade R-CNN (w/o Triplet Loss)	13.4	16.7	40.5	36.6
Cascade R-CNN (Triplet Loss H1 w = 0.050)	12.7	16.1	39.7	35.9

**Table 7:** $MR^{-2}$ results for Faster R-CNN and Cascade R-CNN, comparing the best triplet loss result against the method without it, trained on WiderPedestrian and evaluated on CityPersons.

Method	Reasonable	Small	Heavy	All
Faster R-CNN (w/o Triplet Loss)	15.9	20.9	54.9	44.6
Faster R-CNN (Triplet Loss w = 0.20)	15.6	20.4	53.5	42.7
Cascade R-CNN (w/o Triplet Loss)	16.0	21.6	57.4	-
Cascade R-CNN (Triplet Loss H1 w = 0.150)	13.8	18.1	50.9	39.7

**Table 8:** $MR^{-2}$ values for different state-of-the-art methods evaluated on the CityPersons benchmark.

Method	Reasonable	Small	Heavy
Repulsion Loss [14]	13.2	-	-
ALFNet [12]	12.0	19.0	48.1
CSP (ResNet50) [13]	11.0	16.0	39.4
CSP (HRNet) [20]	9.4	11.4	36.7
Faster R-CNN (VGG16) [49]	15.4	25.6	-
PRNet (ResNet50) [89]	10.8	-	-
BGCNet (HRNet) [92]	8.8	-	-
Faster R-CNN (ResNeXt101) [20]	16.4	-	-
Cascade R-CNN (HRNet) [20]	11.2	14.0	37.1
DA-Net [46]	-	-	51.6
PedJointNet [47]	13.5	-	52.2
PP-Net [48]	12.1	-	53.0

**Table 9:** Results summary for cross-dataset evaluation. All detectors use HRNet as backbone.

Method	Training Set	Test Set	Reasonable	Small	Heavy	All
Cascade R-CNN	WiderPedestrian	CityPersons	16.0	21.6	57.4	-
CSP	WiderPedestrian	CityPersons	17.0	22.4	58.2	-
Ours: Faster R-CNN w. triplet loss (w = 0.1)	WiderPedestrian	CityPersons	15.6	20.4	53.5	42.7
Ours: Cascade R-CNN w. triplet loss (H1 w = 0.15)	WiderPedestrian	CityPersons	13.8	18.1	50.9	39.7

**Table 10:** Results summary for cross-dataset evaluation and progressive training pipeline. All detectors use HRNet as backbone.

Method	Training Set	Test Set	Reasonable	Small	Heavy	All
Cascade R-CNN	CH→ECP	CityPersons	10.3	12.6	40.7	-
Cascade R-CNN	WP→ECP	CityPersons	9.7	11.8	37.7	-
Ours: Cascade R-CNN w. Triplet Loss	WP→ECP	CityPersons	9.9	11.0	36.2	30.8

## Data Availability

No new data were created or analyzed in this study. Data sharing is not applicable to this article.

## References

[1] Girshick R. Fast R-CNN. Proceedings of the IEEE International Conference on Computer Vision.

[2] Ren S., He K., Girshick R., Sun J. (2015). Faster R-CNN: Towards real-time object detection with region proposal networks. Adv. Neural Inf. Process. Syst..

[3] Schroff F., Kalenichenko D., Philbin J. Facenet: A unified embedding for face recognition and clustering. Proceedings of the IEEE Conference on Computer Vision and Pattern Recognition.

[4] Zambrano J.E., Benalcazar D.P., Perez C.A., Bowyer K.W. (2022). Iris recognition using low-level CNN layers without training and single matching. IEEE Access.

[5] Montecino D.A., Perez C.A., Bowyer K.W. (2021). Two-level genetic algorithm for evolving convolutional neural networks for pattern recognition. IEEE Access.

[6] Perez J.P., Perez C.A. (2023). Face Patches Designed through Neuroevolution for Face Recognition with Large Pose Variation. IEEE Access.

[7] Galdames F.J., Perez C.A., Estevez P.A., Adams M. (2022). Rock lithological instance classification by hyperspectral images using dimensionality reduction and deep learning. Chemom. Intell. Lab. Syst..

[8] Perez C.A., Estévez P.A., Galdames F.J., Schulz D.A., Perez J.P., Bastías D., Vilar D.R. Trademark image retrieval using a combination of deep convolutional neural networks. Proceedings of the 2018 International Joint Conference on Neural Networks (IJCNN).

[9] Vilar D.R., Perez C.A. (2021). Extracting structured supervision from captions for weakly supervised semantic segmentation. IEEE Access.

[10] Brazil G., Yin X., Liu X. Illuminating pedestrians via simultaneous detection & segmentation. Proceedings of the IEEE International Conference on Computer Vision.

[11] Zhou C., Yuan J. Bi-box regression for pedestrian detection and occlusion estimation. Proceedings of the European Conference on Computer Vision (ECCV).

[12] Liu W., Liao S., Hu W., Liang X., Chen X. Learning efficient single-stage pedestrian detectors by asymptotic localization fitting. Proceedings of the European Conference on Computer Vision (ECCV).

[13] Liu W., Liao S., Ren W., Hu W., Yu Y. High-level semantic feature detection: A new perspective for pedestrian detection. Proceedings of the IEEE/CVF Conference on Computer Vision and Pattern Recognition.

[14] Wang X., Xiao T., Jiang Y., Shao S., Sun J., Shen C. Repulsion loss: Detecting pedestrians in a crowd. Proceedings of the IEEE Conference on Computer Vision and Pattern Recognition.

[15] Liu Y., Ma J., Wang Y., Zong C. (2020). A novel algorithm for detecting pedestrians on rainy image. Sensors.

[16] Li M., Chen S., Sun C., Fang S., Han J., Wang X., Yun H. (2023). An Improved Lightweight Dense Pedestrian Detection Algorithm. Appl. Sci..

[17] Cao J., Song C., Peng S., Song S., Zhang X., Shao Y., Xiao F. (2020). Pedestrian detection algorithm for intelligent vehicles in complex scenarios. Sensors.

[18] He M., Luo H., Chang Z., Hui B. (2017). Pedestrian detection with semantic regions of interest. Sensors.

[19] Zhang M., Liu Q. (2021). Pedestrian detection by novel axis-line representation and regression pattern. Sensors.

[20] Hasan I., Liao S., Li J., Akram S.U., Shao L. Generalizable pedestrian detection: The elephant in the room. Proceedings of the IEEE/CVF Conference on Computer Vision and Pattern Recognition.

[21] Dollár P., Wojek C., Schiele B., Perona P. Pedestrian detection: A benchmark. Proceedings of the 2009 IEEE Conference on Computer Vision and Pattern Recognition.

[22] Hbaieb A., Rezgui J., Chaari L. Pedestrian detection for autonomous driving within cooperative communication system. Proceedings of the 2019 IEEE Wireless Communications and Networking Conference (WCNC).

[23] Hattori H., Naresh Boddeti V., Kitani K.M., Kanade T. Learning scene-specific pedestrian detectors without real data. Proceedings of the IEEE Conference on Computer Vision and Pattern Recognition.

[24] Huang L., Zhao X., Huang K. Bridging the gap between detection and tracking: A unified approach. Proceedings of the IEEE/CVF International Conference on Computer Vision.

[25] National Highway Traffic Safety Administration (NHTSA) (2023). Overview of Motor Vehicle Traffic Crashes in 2021. https://crashstats.nhtsa.dot.gov/Api/Public/Publication/813435.

[26] European Road Safety Observatory (2023). Facts and Figures—Pedestrians-2023. https://road-safety.transport.ec.europa.eu/system/files/2023-02/ff_pedestrians_20230213.pdf.

[27] Dollár P., Belongie S.J., Perona P. The Fastest Pedestrian Detector in the West. Proceedings of the British Machine Vision Conference.

[28] Li F., Li X., Liu Q., Li Z. (2022). Occlusion handling and multi-scale pedestrian detection based on deep learning: A review. IEEE Access.

[29] Zhang S., Benenson R., Omran M., Hosang J., Schiele B. How far are we from solving pedestrian detection?. Proceedings of the IEEE Conference on Computer Vision and Pattern Recognition.

[30] Liu W., Anguelov D., Erhan D., Szegedy C., Reed S., Fu C.Y., Berg A.C. Ssd: Single shot multibox detector. Proceedings of the European Conference on Computer Vision.

[31] Redmon J., Divvala S., Girshick R., Farhadi A. You only look once: Unified, real-time object detection. Proceedings of the IEEE Conference on Computer Vision and Pattern Recognition.

[32] Cai Z., Vasconcelos N. Cascade R-CNN: Delving into high quality object detection. Proceedings of the IEEE Conference on Computer Vision and Pattern Recognition.

[33] Zhang L., Lin L., Liang X., He K. Is faster R-CNN doing well for pedestrian detection?. Proceedings of the European Conference on Computer Vision.

[34] Zhou K., Liu Z., Qiao Y., Xiang T., Loy C.C. (2023). Domain Generalization: A Survey. IEEE Trans. Pattern Anal. Mach. Intell..

[35] Blanchard G., Deshmukh A.A., Dogan Ü., Lee G., Scott C. (2021). Domain generalization by marginal transfer learning. J. Mach. Learn. Res..

[36] Viola P., Jones M.J., Snow D. (2005). Detecting pedestrians using patterns of motion and appearance. Int. J. Comput. Vis..

[37] Papageorgiou C., Poggio T. (2000). A trainable system for object detection. Int. J. Comput. Vis..

[38] Viola P., Jones M.J. (2004). Robust real-time face detection. Int. J. Comput. Vis..

[39] Dalal N., Triggs B. Histograms of oriented gradients for human detection. Proceedings of the 2005 IEEE Computer Society Conference on Computer Vision and Pattern Recognition (CVPR’05).

[40] Dollár P., Appel R., Belongie S., Perona P. (2014). Fast feature pyramids for object detection. IEEE Trans. Pattern Anal. Mach. Intell..

[41] Felzenszwalb P.F., Girshick R.B., McAllester D., Ramanan D. (2010). Object detection with discriminatively trained part-based models. IEEE Trans. Pattern Anal. Mach. Intell..

[42] He K., Gkioxari G., Dollár P., Girshick R. Mask R-CNN. Proceedings of the IEEE International Conference on Computer Vision.

[43] Angelova A., Krizhevsky A., Vanhoucke V., Ogale A.S., Ferguson D. Real-Time Pedestrian Detection with Deep Network Cascades. Proceedings of the British Machine Vision Conference.

[44] Cai Z., Saberian M., Vasconcelos N. Learning complexity-aware cascades for deep pedestrian detection. Proceedings of the IEEE International Conference on Computer Vision.

[45] Hosang J., Omran M., Benenson R., Schiele B. Taking a deeper look at pedestrians. Proceedings of the IEEE Conference on Computer Vision and Pattern Recognition.

[46] Yin R., Zhang R., Zhao W., Jiang F. (2020). Da-net: Pedestrian detection using dense connected block and attention modules. IEEE Access.

[47] Lin C.Y., Xie H.X., Zheng H. (2019). PedJointNet: Joint head-shoulder and full body deep network for pedestrian detection. IEEE Access.

[48] Cai J., Lee F., Yang S., Lin C., Chen H., Kotani K., Chen Q. (2020). Pedestrian as points: An improved anchor-free method for center-based pedestrian detection. IEEE Access.

[49] Zhang S., Benenson R., Schiele B. Citypersons: A diverse dataset for pedestrian detection. Proceedings of the IEEE Conference on Computer Vision and Pattern Recognition.

[50] Li C., Wang Y., Liu X. (2023). An improved YOLOv7 lightweight detection algorithm for obscured pedestrians. Sensors.

[51] Liu X., Lin Y. (2023). YOLO-GW: Quickly and Accurately Detecting Pedestrians in a Foggy Traffic Environment. Sensors.

[52] Braun M., Krebs S., Flohr F., Gavrila D.M. (2019). Eurocity persons: A novel benchmark for person detection in traffic scenes. IEEE Trans. Pattern Anal. Mach. Intell..

[53] Shao S., Zhao Z., Li B., Xiao T., Yu G., Zhang X., Sun J. (2018). Crowdhuman: A benchmark for detecting human in a crowd. arXiv.

[54] Parkhi O., Vedaldi A., Zisserman A. Deep face recognition. Proceedings of the BMVC 2015—Proceedings of the British Machine Vision Conference 2015.

[55] Trigueros D.S., Meng L., Hartnett M. (2018). Enhancing convolutional neural networks for face recognition with occlusion maps and batch triplet loss. Image Vis. Comput..

[56] Boutros F., Damer N., Kirchbuchner F., Kuijper A. (2022). Self-restrained triplet loss for accurate masked face recognition. Pattern Recognit..

[57] Yeung H.W.F., Li J., Chung Y.Y. Improved performance of face recognition using CNN with constrained triplet loss layer. Proceedings of the 2017 International Joint Conference on Neural Networks (IJCNN).

[58] Feng Y., Wang H., Hu H.R., Yu L., Wang W., Wang S. Triplet distillation for deep face recognition. Proceedings of the 2020 IEEE International Conference on Image Processing (ICIP).

[59] Cheng D., Gong Y., Zhou S., Wang J., Zheng N. Person re-identification by multi-channel parts-based cnn with improved triplet loss function. Proceedings of the IEEE Conference on Computer Vision and Pattern Recognition.

[60] Si T., Zhang Z., Liu S. (2019). Compact triplet loss for person re-identification in camera sensor networks. Ad Hoc Netw..

[61] Fan X., Jiang W., Luo H., Mao W., Yu H. (2020). Instance hard triplet loss for in-video person re-identification. Appl. Sci..

[62] Zhou Q., Zhong B., Lan X., Sun G., Zhang Y., Zhang B., Ji R. (2020). Fine-grained spatial alignment model for person re-identification with focal triplet loss. IEEE Trans. Image Process..

[63] Yin Q., Wang G., Wu J., Luo H., Tang Z. (2022). Dynamic re-weighting and cross-camera learning for unsupervised person re-identification. Mathematics.

[64] Mihaescu R.E., Chindea M., Paleologu C., Carata S., Ghenescu M. (2020). Person Re-identification across data distributions based on general purpose DNN object detector. Algorithms.

[65] Chen W., Chen X., Zhang J., Huang K. Beyond triplet loss: A deep quadruplet network for person re-identification. Proceedings of the IEEE Conference on Computer Vision and Pattern Recognition.

[66] Wang Y., Han C., Yao G., Zhou W. (2021). MAPD: An improved multi-attribute pedestrian detection in a crowd. Neurocomputing.

[67] Lee S.W. Domain generalization with triplet network for cross-corpus speech emotion recognition. Proceedings of the 2021 IEEE Spoken Language Technology Workshop (SLT).

[68] Yu B., Liu T., Gong M., Ding C., Tao D. Correcting the triplet selection bias for triplet loss. Proceedings of the European Conference on Computer Vision (ECCV).

[69] Wang S., Yu L., Li C., Fu C.W., Heng P.A. Learning from extrinsic and intrinsic supervisions for domain generalization. Proceedings of the European Conference on Computer Vision.

[70] Dou Q., Coelho de Castro D., Kamnitsas K., Glocker B. (2019). Domain generalization via model-agnostic learning of semantic features. Adv. Neural Inf. Process. Syst..

[71] Deng W., Zheng L., Sun Y., Jiao J. (2020). Rethinking triplet loss for domain adaptation. IEEE Trans. Circuits Syst. Video Technol..

[72] Dong X., Shen J. Triplet loss in siamese network for object tracking. Proceedings of the European Conference on Computer Vision (ECCV).

[73] Unde A.S., Rameshan R.M. (2021). MOTS R-CNN: Cosine-margin-triplet loss for multi-object tracking. arXiv.

[74] Yin J., Wang W., Meng Q., Yang R., Shen J. A unified object motion and affinity model for online multi-object tracking. Proceedings of the IEEE/CVF Conference on Computer Vision and Pattern Recognition.

[75] Bredin H. Tristounet: Triplet loss for speaker turn embedding. Proceedings of the 2017 IEEE International Conference on Acoustics, Speech and Signal Processing (ICASSP).

[76] Li C., Ma X., Jiang B., Li X., Zhang X., Liu X., Cao Y., Kannan A., Zhu Z. (2017). Deep speaker: An end-to-end neural speaker embedding system. arXiv.

[77] Ren F., Xue S. (2020). Intention detection based on siamese neural network with triplet loss. IEEE Access.

[78] Zhang M., Cheng Q., Luo F., Ye L. (2021). A triplet nonlocal neural network with dual-anchor triplet loss for high-resolution remote sensing image retrieval. IEEE J. Sel. Top. Appl. Earth Obs. Remote. Sens..

[79] Hazra S., Santra A. (2019). Short-range radar-based gesture recognition system using 3D CNN with triplet loss. IEEE Access.

[80] Doras G., Peeters G. A prototypical triplet loss for cover detection. Proceedings of the ICASSP 2020-2020 IEEE International Conference on Acoustics, Speech and Signal Processing (ICASSP).

[81] Sun Z., Hu S., Song H., Liang P. (2023). Learning Wasserstein Contrastive Color Histogram Representation for Low-Light Image Enhancement. Mathematics.

[82] Munder S., Gavrila D.M. (2006). An experimental study on pedestrian classification. IEEE Trans. Pattern Anal. Mach. Intell..

[83] Ess A., Leibe B., Van Gool L. Depth and appearance for mobile scene analysis. Proceedings of the 2007 IEEE 11th International Conference on Computer Vision.

[84] Wojek C., Walk S., Schiele B. Multi-cue onboard pedestrian detection. Proceedings of the 2009 IEEE Conference on Computer Vision and Pattern Recognition.

[85] Loy C.C., Lin D., Ouyang W., Xiong Y., Yang S., Huang Q., Zhou D., Xia W., Li Q., Luo P. (2019). Wider face and pedestrian challenge 2018: Methods and results. arXiv.

[86] Geiger A., Lenz P., Urtasun R. Are we ready for autonomous driving? The kitti vision benchmark suite. Proceedings of the 2012 IEEE Conference on Computer Vision and Pattern Recognition.

[87] Paszke A., Gross S., Massa F., Lerer A., Bradbury J., Chanan G., Killeen T., Lin Z., Gimelshein N., Antiga L. (2019). Pytorch: An imperative style, high-performance deep learning library. Adv. Neural Inf. Process. Syst..

[88] Abadi M., Agarwal A., Barham P., Brevdo E., Chen Z., Citro C., Corrado G.S., Davis A., Dean J., Devin M. (2016). Tensorflow: Large-scale machine learning on heterogeneous distributed systems. arXiv.

[89] Song X., Zhao K., Chu W.S., Zhang H., Guo J. Progressive refinement network for occluded pedestrian detection. Proceedings of the Computer Vision–ECCV 2020: 16th European Conference.

[90] Wang J., Sun K., Cheng T., Jiang B., Deng C., Zhao Y., Liu D., Mu Y., Tan M., Wang X. (2020). Deep high-resolution representation learning for visual recognition. IEEE Trans. Pattern Anal. Mach. Intell..

[91] Dollar P., Wojek C., Schiele B., Perona P. (2011). Pedestrian detection: An evaluation of the state of the art. IEEE Trans. Pattern Anal. Mach. Intell..

[92] Li J., Liao S., Jiang H., Shao L. Box guided convolution for pedestrian detection. Proceedings of the 28th ACM International Conference on Multimedia, Virtual Event.

[93] Cordts M., Omran M., Ramos S., Rehfeld T., Enzweiler M., Benenson R., Franke U., Roth S., Schiele B. The cityscapes dataset for semantic urban scene understanding. Proceedings of the IEEE Conference on Computer Vision and Pattern Recognition.

[94] Chen K., Wang J., Pang J., Cao Y., Xiong Y., Li X., Sun S., Feng W., Liu Z., Xu J. (2019). MMDetection: Open MMLab Detection Toolbox and Benchmark. arXiv.

[95] Simonyan K., Zisserman A. Very Deep Convolutional Networks for Large-Scale Image Recognition. Proceedings of the 3rd International Conference on Learning Representations, ICLR 2015.

